# Quasi‐2D Growth of Aluminum Nitride Film on Graphene for Boosting Deep Ultraviolet Light‐Emitting Diodes

**DOI:** 10.1002/advs.202001272

**Published:** 2020-06-23

**Authors:** Hongliang Chang, Zhaolong Chen, Bingyao Liu, Shenyuan Yang, Dongdong Liang, Zhipeng Dou, Yonghui Zhang, Jianchang Yan, Zhiqiang Liu, Zihui Zhang, Junxi Wang, Jinmin Li, Zhongfan Liu, Peng Gao, Tongbo Wei

**Affiliations:** ^1^ Research and Development Center for Semiconductor Lighting Technology Institute of Semiconductors Chinese Academy of Sciences Beijing 100083 China; ^2^ Center of Materials Science and Optoelectronics Engineering University of Chinese Academy of Sciences Beijing 100049 China; ^3^ Center for Nanochemistry (CNC) Beijing Science and Engineering Center for Nanocarbons College of Chemistry and Molecular Engineering Peking University Beijing 100871 China; ^4^ Beijing Graphene Institute (BGI) Beijing 100095 China; ^5^ Electron Microscopy Laboratory and International Center for Quantum Materials School of Physics Peking University Beijing 100871 China; ^6^ State Key Laboratory of Superlattices and Microstructures Institute of Semiconductors Chinese Academy of Sciences Beijing 100083 China; ^7^ School of Electronics and Information Engineering Hebei University of Technology Tianjin 300401 China; ^8^ Collaborative Innovation Center of Quantum Matter Beijing 100871 China

**Keywords:** aluminum nitride, deep ultraviolet light‐emitting diodes, graphene, quasi‐2D growth

## Abstract

Efficient and low‐cost production of high‐quality aluminum nitride (AlN) films during heteroepitaxy is the key for the development of deep ultraviolet light‐emitting diodes (DUV‐LEDs). Here, the quasi‐2D growth of high‐quality AlN film with low strain and low dislocation density on graphene (Gr) is presented and a high‐performance 272 nm DUV‐LED is demonstrated. Guided by first‐principles calculations, it is found that AlN grown on Gr prefers lateral growth both energetically and kinetically, thereby resulting in a Gr‐driven quasi‐2D growth mode. The strong lateral growth mode enables most of dislocations to annihilate each other at the AlN/Gr interface, and therefore the AlN epilayer can quickly coalesce and flatten the nanopatterned sapphire substrate. Based on the high quality and low strain of AlN film grown on Gr, the as‐fabricated 272 nm DUV‐LED shows a 22% enhancement of output power than that with low‐temperature AlN buffer, following a negligible wavelength shift under high current. This facile strategy opens a pathway to drastically improve the performance of DUV‐LEDs.

## Graphical abstract

High‐quality aluminum nitride (AlN) film with low strain and low dislocation density is realized by graphene (Gr)‐driven quasi‐2D growth mode. Thus‐obtained AlN film enables much better photoelectric performance of the as‐fabricated deep ultraviolet light‐emitting diode (DUV‐LED) devices, which demonstrates practical applications for directly grown Gr film and may bring the disruptive technology for the growth of high‐quality AlN and high efficiency of DUV‐LEDs.

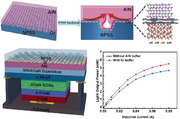

AlGaN‐based deep ultraviolet light‐emitting diodes (DUV‐LEDs) have received a wide attention owing to their broad application prospects in the fields of sterilization, polymer curing, biochemical detection, non‐line‐of‐sight communication, and special lighting.^[^ ^1^, ^2^, ^3^, ^4^, ^5^ ^]^ Due to lack of cost‐effective homogeneous substrate, aluminum nitride (AlN) is usually heteroepitaxially grown on the sapphire substrate, which results in a large number of dislocations in the AlN epilayer and severely deteriorates the properties of DUV‐LEDs.^[^ ^6^, ^7^, ^8^, ^9^ ^]^ To improve the quality of AlN films grown on sapphire, various methods have been implemented including NH_3_ pulsed‐flow technique and epitaxial lateral overgrowth on patterned sapphire substrates and patterned AlN/sapphire templates.^[^ ^10^, ^11^, ^12^, ^13^ ^]^ Among the current state‐of‐the‐art technology, the nanopatterned sapphire substrate (NPSS) is widely regarded as a stable and effective method to achieve epitaxial growth of high‐quality AlN films and high‐performance DUV‐LEDs.^[^ ^14^, ^15^, ^16^, ^17^ ^]^ However, due to the high surface adhesion coefficient of Al atom on sapphire (or NPSS), the growth of AlN usually follows a 3D Volmer–Weber growth mode driven by the surface energy constraint.^[^ ^18^, ^19^, ^20^ ^]^ Under Volmer–Weber mode, high density of dislocations is generated during the island coalescence at the AlN/NPSS interface, which easily propagate upward to the upper epilayer. In addition, 3D island growth also leads to a much higher coalescence thickness (over 3 µm) on NPSS than on flat sapphire (FS) as long growth time is required to cover the pattern^[^ ^17^ ^]^ and thus high cost. The key to changing the conventional 3D growth mode is to alleviate the strong adsorption of large mismatched substrate to the epilayer, and 2D material as a buffer layer, which can shield the substrate to some extent, and is definitely a method worth considering.^[^ ^21^, ^22^, ^23^ ^]^

Graphene (Gr), as an ideal 2D material buffer layer, has been proposed to effectively reduce the mismatch effects between nitride and substrate due to weak van der Waals bonds and further helps to achieve transferable optoelectronics and electronics devices.^[^ ^24^, ^25^, ^26^, ^27^, ^28^ ^]^ Thus, the direct growth of group‐III nitride epilayers on Gr by quasi‐van der Waals epitaxy (QvdWE) can relax the strict requirement of the conventional heteroepitaxy. More notably, Gr can not only reduce the surface migration barrier during QvdWE growth of nitride, but also promote the lateral migration of metal Al atoms, boosting the 2D mode growth.^[^ ^29^, ^30^ ^]^ Al Balushi et al. have already demonstrated the synthesis of 2D GaN via a migration‐enhanced encapsulated growth technique utilizing epitaxial Gr.^[^ ^31^ ^]^ However, the essential role of Gr as a buffer layer to promote the 2D growth of AlN is still ambiguous. Moreover, QvdWE growth of AlN films on Gr to improve DUV‐LED devices remains relatively unexplored, compared to mature blue LEDs.^[^ ^17^, ^32^ ^]^

Here, we successfully realize QvdWE growth of high‐quality AlN film with low stress and low dislocation density in a quasi‐2D growth mode on NPSS using Gr as the buffer layer and demonstrate its application in high‐performance DUV‐LED. By first‐principles calculations, we confirm that AlN on Gr is more prone to 2D lateral growth in terms of energy and kinetics and clearly elucidate the growth model. Meanwhile, with the help of the Gr‐driven quasi‐2D growth, the AlN film can rapidly coalesce and cover the concave‐cone NPSS below a thickness of about 1 µm. The strong lateral growth also enables lots of dislocations to annihilate each other at the AlN/Gr interface. Finally, the 272 nm DUV‐LED with Gr exhibits increased output power and decreased reverse leakage, compared to that with conventional low‐temperature (LT) AlN buffer. Our work demonstrates practical applications for directly grown Gr film that may bring the disruptive technology for the growth of high‐quality AlN and high efficiency of DUV‐LEDs.

Above all, in order to compare the growth modes of AlN on Gr and Al_2_O_3_ surfaces, we study the adsorption and diffusion of an Al atom on small AlN clusters on the two substrates from first‐principles. We adopt Vienna ab initio simulation package^[^ ^33^ ^]^ and calculate the diffusion barrier using the nudged elastic band method.^[^ ^34^ ^]^ All the other computational details can be found in the previous paper.^[^ ^35^ ^]^ First, we consider the AlN growth on Al_2_O_3_ (0001) surface. The most stable surface is Al‐terminated, and one‐third of Al sites (low‐Al sites) have been occupied. The adsorption of Al on high‐Al sites is more stable than the vac‐Al sites by 1.02 eV.^[^ ^35^ ^]^ We add four Al atoms (three at high‐Al sites and one at the vac‐Al site) and three N atoms to form a small Al_10_N_3_ cluster on the Al_2_O_3_ 3 × 3 surface cell (see the black triangle in **Figure** **1**a), and then introduce another Al atom to the cluster. The most stable adsorption site on the Al_2_O_3_ surface and near the edge of the AlN cluster is the nearby high‐Al site, as shown by the solid red circle in Figure 1a. However, the top site on N atom is more stable by 0.085 eV. Despite the small energy difference, the diffusion barrier from the top site to the high‐Al site is calculated to be 1.107 eV (Figure 1b). Therefore, the coming Al atom is energetically favorable to adsorb on the top of the existing AlN cluster and is difficult to jump down to the Al_2_O_3_ surface. In other words, the AlN on Al_2_O_3_ surface prefers a 3D growth mode. Only at relatively high growth temperature, the Al atom on the top site can overcome the large energy barrier and jump down to the Al_2_O_3_ surface, facilitating the lateral growth of AlN.

**Figure 1:**
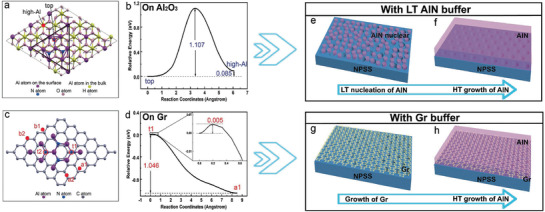
First‐principles calculation and schematic diagram of the growth of AlN on Al_2_O_3_ and Gr. a) Adsorption sites of Al atom to the Al_10_N_3_ cluster on Al_2_O_3_ (0001) surface. The Al_10_N_3_ cluster is denoted by a triangle. The solid red circle represents the high‐Al adsorption site that is on the Al_2_O_3_ surface and next to the Al_10_N_3_ cluster, while the empty red circle represents the top site on top of an N atom in the Al_10_N_3_ cluster. The H atoms are used to passivate the bottom layer of the Al_2_O_3_ slab. b) The energy barrier of Al atom diffusing from the top site to the high‐Al site calculated from NEB method. c) Adsorption sites of Al atom to the Al_9_N_4_ cluster on Gr surface. The solid red circles (a and b sites) represent the Al adsorption sites that are on the Gr surface and next to the Al_9_N_4_ cluster, while the empty red circle represents the top site on top of N atoms in the Al_9_N_4_ cluster. d) The energy barrier of Al atom diffusing from the t1 site to the a1 site calculated from NEB method. The small energy barrier is enlarged in the insect. e–h) Schematic diagram of the key processes of growing AlN film on NPSS with a LT AlN or Gr buffer, including e) LT nucleation growth of AlN, f) HT growth of AlN film, g) direct growth of Gr films on NPSS, and h) one‐step HT growth of AlN film.

However, we find a different growth mode on the Gr sheet. We calculate the Al adsorption on a small Al_7_N_3_ cluster on Gr 6 × 6 cell (Figure S1, Supporting Information), and find that the adsorption of the additional Al atom on top of the N atom is unstable. Instead, the Al atom will jump down to the Gr sheet, attaching to the edge of the Al_7_N_3_ cluster. This clearly indicates that the lateral growth is preferred for small AlN cluster on Gr. Furthermore, we considered the adsorption of Al atom on a larger Al_9_N_4_ cluster. Two top sites are found to be stable with adsorption energies around 1.8–1.9 eV, as shown by t1 and t2 sites in Figure 1c. The top sites on the other two N atoms are unstable as in the above Al_7_N_3_ case. On the Gr sheet, there are two types of adsorption sites near the edge of the Al_9_N_4_ cluster. As shown in Figure 1c, one type is the a1 and a2 sites next to the N atoms in the cluster, and the other type is the b1 and b2 sites without nearby N atoms. The Al adsorption on b1 and b2 sites is slightly more favorable than the top sites, with adsorption energies of 1.9–2.1 eV. However, the most stable adsorption sites for Al atom is the a1 and a2 sites, with considerable larger adsorption energies of 2.7–2.9 eV. Moreover, the energy barrier from the top t1 site to the lateral a1 site is calculated to be only 0.005 eV (Figure 1d). Considering such a tiny barrier, the Al atom on the t1 site is quite easy to jump down to the Gr surface. Therefore, we conclude that the AlN on Gr prefers lateral 2D growth both energetically and kinetically at the initial growth stage as quasi‐2D mode, which is expected to be verified in experiments. For the purpose of verifying the authenticity of the results obtained by first‐principles calculations, experiments of AlN growth on NPSS with different buffer layers (LT AlN buffer layer or Gr buffer layer) are designed. Figure 1e,f and Figure 1g,h schematically show the key processes of growing AlN film using LT AlN and Gr as buffer layer, following the high‐temperature (HT) growth of AlN epilayers. It is named as with LT AlN buffer and with Gr buffer, respectively.

**Figure** **2**a shows the scanning electron microscopy (SEM) images of the utilized NPSS surface, and the cross‐sectional profile of the patterns is illustrated as the inset. The period of NPSS pattern is 1 µm, and the depth and width of the holes are 300 and 300 nm, respectively. In order to ensure that AlN can be high‐quality epitaxial growth on a uniform and continuous Gr film buffered NPSS, we directly grow bilayer Gr on two‐inch NPSS by atmospheric‐pressure chemical vapor deposition method.^[^ ^36^ ^]^ Figure S2, Supporting Information, shows an image of as‐grown two‐inch Gr/NPSS wafer, and we can see that the NPSS is fully covered with uniform Gr (Figure 2b). The synthesized Gr is characterized by Raman spectra. The black curve in Figure 2c is the typical Raman spectra of grown Gr, which displays the characteristic Raman peaks of Gr (D‐1340 cm^−1^, G‐1587 cm^−1^, and 2D‐2682 cm^−1^). Moreover, Raman mapping (with laser spot around 1 µm and step 1 µm) results confirm that the synthesized Gr is relatively uniform at micron‐scale (Figure 2d–g). Furthermore, as shown in Figure 2h, due to the absorption of ultraviolet light by Gr itself,^[^ ^37^ ^]^ the characterized transmittance spectrum of Gr/NPSS in the wide spectral range (200–850 nm) shows an absorption peak in the ultraviolet band. Even so, Gr/NPSS still maintains a decent light transmittance of ≈83% in the target band of the intended LED (272 nm). To enhance the reactivity of Gr for AlN nucleation, Gr covered NPSS is implemented by N_2_ plasma treatment prior to all AlN growth experiments on Gr/NPSS in this work. It is known that the concentration of defect in Gr can be reflected by the *I*_D_/*I*_G_ from the Raman result.^[^ ^27^ ^]^ It is clear from Figure 2c that after plasma treatment, the *I*_D_/*I*_G_ of Raman spectrum is enhanced, which is an indication of the increased amount of dangling bonds. Thus, the directly grown Gr on NPSS is highly uniform and can serve as an ideal epitaxial substrate after plasma treatment.

**Figure 2:**
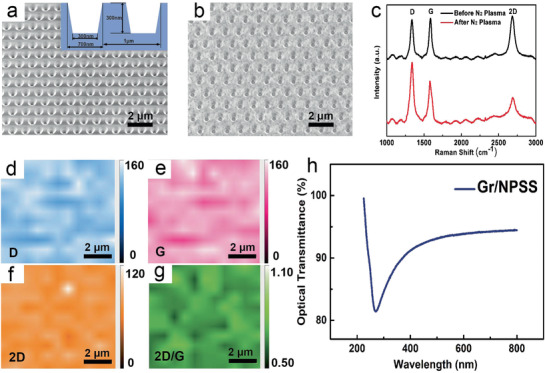
SEM, Raman, and optical transmission spectra analyses of bare and Gr covered NPSSs. a) Bird's eye view SEM image of the NPSS surface with nanoconcave‐cone pattern under a tilt angle of 25°. The cross‐sectional profile of the patterns of NPSS is illustrated in the inset in (a). b) SEM image of the 25° tilted surface of the as‐grown Gr layers on NPSS. c) Raman spectra of Gr on NPSS before N_2_ plasma treatment (black) and after N_2_ plasma (red). d–g) Raman mapping of d) D‐peak, e) 2D peak, f) G‐peak, and g) *I*_2D_/*I*_G_ ratio on a Gr film in a 10 × 10 µm^2^ region. h) Optical transmission spectrum of Gr/NPSS.

Experiments of epitaxial growth of AlN on FS are performed to initially verify the above‐mentioned quasi‐2D mode obtained by first‐principles calculations. From **Figure** **3**a_1_–a_3_, we can see that the epitaxy growth of AlN on FS with Gr coalesces quickly after nucleation at high density, resulting in quasi‐flat surface morphology with a thickness of only 150 nm and a mirror‐smooth surface with 500 nm. The rapid lateral coalescence of AlN in the presence of Gr might be attributed to the fact that Gr can effectively promote a strong 2D growth tendency throughout the AlN growth process, consistent with the expected quasi‐2D results of the first‐principles calculations. Quasi‐2D growth of AlN on Gr from the initial stage enables the dense AlN nuclei to coalesce quickly and to keep smooth film. We further carry out the AlN growth on NPSS with nanoconcave‐cone pattern using metal organic chemical vapor deposition (MOCVD) by the following procedure. According to conventional two‐step growth, a 60 nm LT AlN buffer is grown at 650 °C for 6 min (Figure 3b_1_). For comparison, one‐step growth of HT AlN is also carried out directly on NPSS with Gr buffer at 1270 °C for 6 min (Figure 3c_1_). Then, AlN epilayers are further grown on the aforementioned two templates with different buffer layers at 1270 °C for 2 h to fully flatten the NPSS (Figure 3b_2_,c_2_).

**Figure 3:**
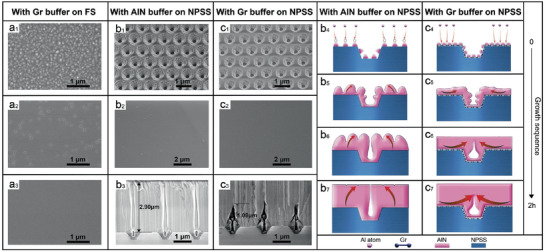
SEM characterization and schematic diagrams of AlN films grown with different buffer layers. a_1_–a_3_) SEM images of a_1_) 30 nm, a_2_) 150 nm, and a_3_) 500 nm AlN grown at 1270 °C on flat sapphire with Gr buffer. b_1_) SEM image of AlN grown on NPSS for 6 min at 650 °C. c_1_) SEM image of AlN directly grown for 6 min at 1270 °C on NPSS with Gr buffer. b_2_,c_2_) The SEM images of AlN grown on NPSS at a HT (1270 °C) for 2 h with b_2_) LT (650 °C) AlN buffer and c_2_) Gr buffer, respectively. b_3_,c_3_) The cross‐sectional SEM images of AlN films grown on NPSS at a HT (1270 °C) for 2 h with b_3_) LT (650 °C) AlN buffer and c_3_) Gr buffer, respectively. b_4_–b_6_,c_4_–c_6_) Schematic illustration of morphology evolution for AlN films grown with b_4_–b_6_) LT AlN buffer and c_4_–c_6_) Gr buffer, respectively.

The initial growth morphology of the two growth processes is compared to reveal the buffer influence. The LT AlN buffer grown for 6 min has dense but uniform smaller nucleation islands (Figure 3b_1_), and a flat film surface is obtained after continued HT growth for 2 h (Figure 3b_2_). In contrast, the QvdWE growth of AlN with Gr buffer at 1270 °C for 6 min exhibits a surprisingly continuous surface morphology in the flat region of NPSS (Figure 3c_1_), further proving that AlN does indeed grow on the Gr surface with quasi‐2D mode. After growing for 2 h, AlN film demonstrates an extremely smooth surface with root‐mean‐square roughness of only 0.142 nm (Figure 3c_2_ and Figure S3c, Supporting Information). It is noted that the thickness of the lateral coalescence of AlN grown on NPSS with LT AlN buffer is 2.90 µm, as depicted in Figure 3b_3_, which are consistent with the conventional Volmer–Weber model of nitride growth on NPSS.^[^ ^15^, ^17^ ^]^ In sharp contrast, the AlN film with Gr buffer has a lateral coalescence thickness of only 1.08 µm from the SEM images of the cross section (Figure 3c_3_), attributed to the Gr‐driven quasi‐2D growth mode of AlN film. For a comparison, the HT AlN growth on NPSS without buffer demonstrates rough and nonuniform surface morphology after 2 h growth (see details in Figure S3, Supporting Information).

Based on the above calculations and SEM characterizations, we propose a model to elucidate the effect of different buffer layers on the growth of AlN from cluster coalescence to covering pattern until the film is formed. As is shown in Figure 3b_4_, at the initial stage of AlN growth on NPSS at LT, Al atoms are easily adsorbed above the as‐grown AlN cluster due to the minimum energy principle. At the same time, the LT condition reduces the diffusion length of the Al adatoms so that the Al atoms tend to be stably adsorbed in the lowest energy position. It means above the grown AlN cluster (Figure 3b_4_), there is a typical 3D‐dominated Volmer–Weber growth mode of AlN (Figure 3b_5_). Therefore, the lateral coalescence tendency of AlN clusters is relatively slow and it is hard to quickly cover the concave‐cone pattern of NPSS, as is shown in Figure 3b_6_,b_7_. In contrast, the introduction of the Gr buffer avoids the problems encountered in the above growth process. On the surface of the Gr treated by N_2_ plasma, AlN can nucleate relatively densely and uniformly at HT. In the presence of the Gr buffer, Al adatoms achieve high enough mobility at HT to move to energetically favorable sites, which is the edge of the nuclear cluster (Figure 3c_4_). Meanwhile, compared to sapphire, Al adatoms have a lower migration barrier on the Gr surface, which means that Al adatoms can easily diffuse on the Gr surface and have a longer lateral diffusion length.^[^ ^35^ ^]^ Therefore, the growth mode of the epitaxial growth of AlN on Gr/NPSS is not the 3D‐dominant Volmer–Weber mode, but a quasi‐2D growth mode with lateral expansion as the main trend from the initial stage (Figure 3c_5_). Thus, it enables AlN to more rapidly coalesce and cover the concave‐cone pattern (Figure 3c_6_) and finally form an extremely smooth film (Figure 3c_7_).

Transmission electron microscopy (TEM) and Raman spectrum are used to reveal the underlying effect mechanism of different buffer layers on AlN crystal during the epitaxial process on NPSS in depth. Dark‐field (DF) TEM images of the interface with LT AlN buffer and Gr buffer by using the reflection *g* = [0002] are presented in **Figures** **4**a,e, respectively. The areas enclosed with white lines are the underlying nanopatterns of the sapphire, above which the air voids are formed during the growing process. From the TEM analysis, we find that the dislocations of AlN films are mostly located on the flat surface of the NPSS between the concave‐cone patterns rather than above the pattern. As for the AlN film grown with LT AlN buffer (Figure 4a), we can distinctly observe a dislocation‐intensive layer on the flat surface. Magnified images of the blue rectangular region further confirm that the dislocation‐dense region has a thickness of about 60 nm, which is consistent with the thickness of the LT AlN buffer (Figure 4b). Therefore, there is no ambiguity that the dislocation‐intensive area is actually the polycrystalline layer formed by the disordered growth of AlN in the LT nucleation stage. Meanwhile, lots of dislocations in the polycrystalline layer are not annihilated in the subsequent lateral growth but spread upward, resulting in a surface etch pit density (EPD) dislocation density of 1.7 × 10^8^ cm^−2^ estimated by etching process in mixed acid solution (see details in Figure S4a, Supporting Information), which could severely degrade the device properties fabricated on it. Luckily, Gr buffer can significantly improve this situation. The high‐resolution TEM (HRTEM) image in Figure 4c exhibits a sharp interface of AlN/Gr/Al_2_O_3_ between the concave‐cone patterns, which confirms the stable existence of layered Gr throughout the growth process. And the cross‐sectional atomic structure of AlN film QvdWE on N_2_ plasma‐treated Gr/Al_2_O_3_ is shown schematically in Figure 4d. From Figure 4e,f, we can recognize that the existence of Gr introduces multiple effects. On the one hand, the number of dislocations produced at the AlN/Gr/Al_2_O_3_ interface between the concave‐cone patterns is greatly reduced. On the other hand, the growth of Gr‐driven AlN presents a strong 2D trend, which leads to some dislocations to incline and stacking faults to annihilate without extending to the surface of the film. Therefore, by taking the advantage of 2D growth process, the estimated surface EPD of the AlN epilayer grown with Gr is reduced to 8.3 × 10^7^ cm^−2^ observed in the same etching experiment mentioned above by SEM, as depicted in Figure S4b, Supporting Information. Meanwhile, the dislocation densities of AlN films with LT AlN and with Gr buffer measured by X‐ray rocking curve are 3.264 × 10^9^ and 2.755 × 10^9^ cm^−2^, respectively, which further proves the better crystal quality of AlN film with Gr (see details in Figure S5, Supporting Information). Hence, the quality improvement of AlN film should be attributed to the fact that Gr alleviates the mismatch effects between the substrate and AlN film to a certain extent. More importantly, the Gr‐driven quasi‐2D growth causes few defects generated at the AlN/Gr/NPSS interface, preventing the propagation of dislocations toward the epitaxial film surface. Also, the selected‐area electron diffraction (SAED) pattern of the AlN domain shows a single diffraction pattern of the (0002) *c*‐axis‐oriented wurtzite structure and the SAED pattern from the interface region proves the epitaxial orientation relationships of (0002) AlN//(0006) Al_2_O_3_ and (01$\bar{1}$0) AlN//(1$\bar{2}$10) Al_2_O_3_ (Figure S6, Supporting Information).

**Figure 4:**
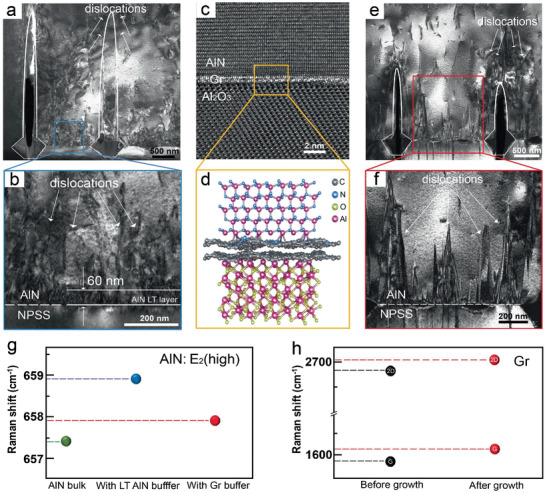
Cross‐sectional TEM and Raman characterizations of AlN films grown on NPSS with the different buffer layers. a,e) DF images of epitaxial AlN on NPSS with a) LT AlN buffer and e) Gr buffer with *g* = [0002]. b,f) Magnified DF images of the AlN/NPSS interface in the blue rectangular region marked in (a) and the AlN/Gr/NPSS interface in the red rectangular region marked in (e). c) The HRTEM image of the AlN/Gr/NPSS interface. d) The schematic diagram of AlN/Gr/Al_2_O_3_ sandwich structure. g) Relative Raman shifts of E_2_ (high) of AlN in stress‐free AlN bulk, AlN/NPSS, and AlN/Gr/NPSS, respectively. h) Relative Raman shifts of G and 2D peaks of Gr before and after AlN growth.

Furthermore, the strains in AlN could impose profound effects on its E_2_ (high) phonon mode of Raman spectra, which could provide a clear signature for the biaxial stress within the basal plane.^[^ ^38^, ^39^ ^]^ With the Gr buffer, the E_2_ (high) peak of AlN is located at 657.9 cm^−1^, which is very close to the stress‐free AlN (657.4 cm^−1^) as shown in Figure 4g. However, the AlN film grown with LT AlN buffer (658.9 cm^−1^) demonstrates higher frequency due to suffering a high compressive strain.^[^ ^38^ ^]^ Compared with the residual stress in AlN with LT AlN buffer (0.42 GPa), the residual compressive stress of AlN with Gr buffer (0.14 GPa) is significantly reduced.^[^ ^40^ ^]^ Meanwhile, the 2D and G peaks of Raman spectrum of Gr also exhibit high stain sensitivity.^[^ ^41^ ^]^ As shown in Figure 4h, G and 2D peaks of the Raman spectrum of Gr sandwiched between AlN and NPSS shift to higher wave numbers (1612.3 and 2705.3 cm^−1^, respectively), with regard to that of the pristine ones before growth (1587.6 and 2682.9 cm^−1^, respectively), which implies that Gr is subjected to compressive stress. Therefore, we can infer that Gr relaxes the stress caused by the mismatch between epilayer and substrate after the combination of Gr distortion of itself (Figure 4d), so that AlN with Gr exhibits a weak residual stress state.

Finally, the DUV‐LED structures are grown on AlN templates on NPSS with different buffer layers and the fabricated DUV‐LED devices are further investigated. The schematic chip structure is shown in **Figure** **5**a, consisting of a 100 nm p‐GaN hole injection layer, an 80 nm thick layer of Mg‐doped p‐AlGaN (p‐Al_0.5_Ga_0.5_N (30 nm) cladding layer and 50 nm thick layer of Mg‐doped p‐Al_0.65_Ga_0.35_N electron blocking layer (EBL)), five‐period Al_0.4_Ga_0.6_N (3 nm)/Al_0.5_Ga_0.5_N (12 nm) multiquantum wells (MQWs), 1.8 µm thick Si‐doped n‐Al_0.55_Ga_0.45_N, 20‐period AlN (20 nm)/Al_0.6_Ga_0.4_N (20 nm) superlattice (SL), and 3‐µm AlN. The low magnification cross‐sectional scanning TEM image of the DUV‐LED grown on the Gr/NPSS‐based AlN template (Figure S7, Supporting Information) and corresponding energy dispersive spectroscopy mapped images (Figure S8, Supporting Information) clearly show the DUV‐LED heterojunction structure. Figure 5b and Figure S9, Supporting Information, plot the current–voltage characteristics of the DUV‐LEDs on NPSS with different buffer layers and without buffer layer, respectively. Compared with the LT AlN buffered DUV‐LED with a turn‐on voltage of 6.1 V, the DUV‐LED with Gr buffer shows a slight increase of 6.5 V. It is worth noting that with the increase of applied reverse voltage, as‐fabricated DUV‐LED with Gr consistently shows the much lower reverse leakage current in Figure 5c (only 0.02 µA at −8 V), confirming the reduced dislocation density of the DUV‐LED with Gr buffer. In comparison, the reverse leakage current of DUV‐LED with LT AlN buffer reaches 0.12 µA at −8 V, while the DUV‐LED without buffer exhibits large reverse leakage current even at low reverse voltage (10^−3^ A at −8 V; Figure S9b, Supporting Information).

**Figure 5:**
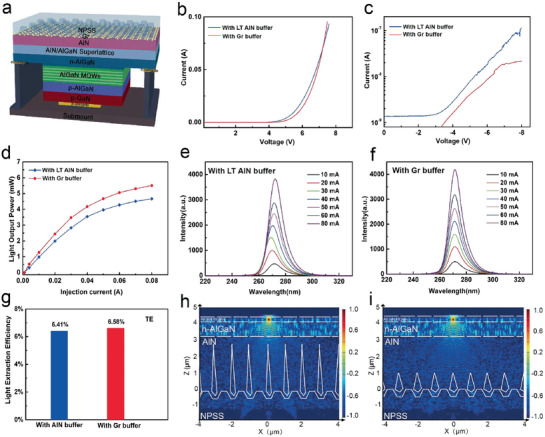
The EL and FDTD simulation characterization of as‐fabricated DUV‐LEDs. a) Schematic illustration of the DUV‐LEDs structure with Gr. b) Current–voltage characteristics of as‐fabricated DUV‐LEDs with different buffer layers. c) The reverse current–voltage curves on a semi‐log scale. d) The light output power of the as‐fabricated DUV‐LEDs with different buffer layers as a function of injection current. e,f) EL spectra of different DUV‐LEDs by varying the injection current from 10 to 80 mA. g) FDTD simulations of the LEE of DUV‐LEDs with different buffer layers for TE polarizations. h,i) Cross‐sectional electric field distributions of the DUV‐LEDs with h) LT buffer and i) Gr buffer for TE polarizations at the *x*–*z*‐plane, respectively.

The light output power (LOP) of 272 nm DUV‐LEDs as a function of injection current is plotted in Figure 5d and Figure S10a, Supporting Information. The LOP of LEDs increases simultaneously with increasing the injection current and the LOP of the LED with Gr is obviously higher than those with LT‐AlN buffer and without buffer due to the improved crystal quality. At 20 mA, the DUV‐LED with Gr buffer offers a LOP of 2.43 mW, which is higher than that of the DUV‐LED with LT AlN buffer (1.99 mW), and is about 4.2 times the LOP of the DUV‐LED without the buffer (0.58 mW) (Figure S10a, Supporting Information). According to the calculation, when the applied current is 20 mA, the corresponding external quantum efficiency (EQE) and wall‐plug efficiency (WPE) of DUV‐LED with Gr buffer layer are 2.66% and 1.94%, respectively, which are both higher than the EQE (2.17%) and WPE (1.65%) of LED with LT AlN buffer layer. In order to evaluate the reliability, the normalized electroluminescence (EL) of as‐fabricated LEDs under different injection currents is investigated as shown in Figure 5e,f and Figure S10b, Supporting Information. The EL peak wavelength of the DUV‐LED using Gr as buffer shows a 0.8 nm blue‐shift as the applied current increases from 10 to 80 mA, obviously reducing the wavelength shift compared to that of the LED with LT AlN buffer (2.4 nm) and without buffer (5.5 nm). It is attributed to substantial stress relaxation due to the presence of Gr buffer layer, while the epitaxial high‐quality LED structure with Gr reduces the non‐radiative recombination normally associated with device heating.

Moreover, the light extraction efficiency (LEE) of the DUV‐LEDs with different buffer layers is numerically calculated by using a 3D finite difference time domain (FDTD) method. The air void size and height are obtained from cross‐sectional TEM and SEM results. From simulation results, we find that TE‐ and TM‐polarized LEEs in the DUV‐LED with LT AlN buffer layer (6.41% and 0.53%, respectively) and with Gr buffer layer (6.58% and 0.57%, respectively) are almost same (Figure 5g and Figure S11, Supporting Information). Meanwhile, the TE‐ and TM‐polarized cross section electric field distributions for DUV‐LEDs with different buffers are analogical (Figure 5h,i and Figure S12, Supporting Information). These simulation results indicate that the insertion of Gr changes the shape of air void in the AlN film, but does not deteriorate LEE of DUV‐LED. Therefore, the efficiency improvement of DUV‐LED with Gr buffer is mostly attributed to the reduced dislocation density and strain in the epilayers, corresponding to the enhancement of internal quantum efficiency (IQE).

In summary, our accomplishments, quasi‐2D growth of high‐quality AlN film directly realized on Gr‐buffered NPSS and demonstrates excellent optoelectronic properties and reliability of DUV‐LED. Guided by the first‐principles calculations, we reveal that AlN on Gr tends to 2D lateral growth energetically and kinetically. Meanwhile, confirmed by experiments, the growth mode of AlN on Gr‐buffered NPSSS is indeed dominated by a quasi‐2D growth, different from the traditional 3D‐dominated Volmer–Weber growth on bare NPSS. Thus, effective improvement of the AlN crystal quality is realized by facilitating mutual annihilation of the dislocations during quasi‐2D growth process, following significant reduction in the coalescence thickness of AlN. Eventually, a series of EL characterization and simulation studies emphasize that the output power of DUV‐LED with Gr exhibits a 22% enhancement compared to its counterpart with LT AlN buffer. Through both experimental and theoretical analysis, it is concluded that the Gr buffer opens a new pathway to drastically improve the performance of DUV‐LEDs, as a disruptive technology.

## Experimental Section

##### CVD Growth of Gr on NPSS

Typically, the two‐inch NPSS was cleaned with deionized water, ethanol, and acetone and then loaded into a three‐zone HT furnace. The furnace was heated to 1050 °C and stabilized for about 10 min under 500 sccm Ar and 300 sccm H_2_. 30 sccm CH_4_ was introduced into the reaction chamber as carbon source for the growth of Gr on the NPSS for about 3–5 h.

##### MOCVD Growth of AlN on NPSS with Gr Buffer Layer

The Gr/NPSS was exposed to N_2_ plasma treatment (PVA TePla AG, 300 Standard) under optimized plasma treatment conditions (200 Pa pressure with 300 sccm air flow and 50 w power for 30 s) before loading into MOCVD chamber.

The HT AlN was grown at 1270 °C for 2 h with an NH_3_ flow of 500 sccm and a trimethylaluminum (TMAl) flow of 70 sccm. It was a one‐step process without using LT buffer layer and the growth rate for the AlN epilayer was 1.5 µm h^−1^.

##### MOCVD Growth of DUV‐LED Structure on Gr/NPSS

The MOCVD system used in the epitaxial growth process of DUV‐LEDs was a home‐made vertical single‐chip system. The AlGaN‐based DUV‐LED structure was grown on the AlN/Gr/sapphire template, including a 20‐period AlN/Al_0.6_Ga_0.4_N SL, n‐Al_0.55_Ga_0.45_N layer, five‐period Al_0.4_Ga_0.6_N/Al_0.5_Ga_0.5_N MQWs, and p‐type layers (layer of Mg‐doped p‐Al_0.65_Ga_0.35_N EBL, p‐Al_0.5_Ga_0.5_N cladding layer and p‐GaN contact layer). Trimethylgallium (TMGa) was used as a Ga precursor. Silane (SiH_4_) and bis(cyclopentadienyl)magnesium (Cp_2_Mg) were used for n‐type and p‐type doping, respectively. A 20‐period AlN (2 nm)/Al_0.6_Ga_0.4_N (2 nm) SL was first deposited at 1130 °C, with the periodic flow change of TMAl to adjust the deposition component while the TMGa flow was kept at 32 sccm. Then temperature was reduced to 1002 °C, and 20 sccm silicane‐hydrogen mixture flow (actual silicane flow rate was 2.34 sccm) was introduced for the growth of the 1.8 µm n‐Al_0.55_Ga_0.45_N layer. The five‐period Al_0.5_Ga_0.5_N/Al_0.4_Ga_0.6_N MQWs was further grown with a 3 nm quantum well and a 12 nm quantum barrier by switching the TMAl from 24 to 14 sccm and TMGa from 8 to 7 sccm for each period. A 50 nm thick layer of Mg‐doped p‐Al_0.65_Ga_0.35_N EBL, a p‐Al_0.5_Ga_0.5_N (30 nm) cladding layer, and a 100 nm thick p‐GaN contact layer were subsequently deposited. After the growth, the p‐type layers were annealed in the reactor at 800 °C in N_2_ atmosphere for 20 min to activate the Mg acceptors. In addition, the doping concentration of Si in the n‐Al_0.55_Ga_0.45_N layer was 3 × 10^18^ cm^−3^ and the doping concentration of Mg in p‐GaN was 1 × 10^18^ cm^−3^. Moreover, the corresponding estimated carrier concentrations of n‐type layer and p‐type layer were 3 × 10^18^ and 1 × 10^16^ cm^−3^, respectively.

##### DUV‐LED Device Fabrication

DUV‐LED devices with a die size of 0.5 mm × 0.5 mm were fabricated with the standard LED processes of photolithography, ICP etching, and e‐beam evaporation. A Ti/Al/Ti/Au metal stack was deposited on the exposed n‐AlGaN as the n‐type contact, and a Ni/Au stack was used as the p‐type contact. Finally, the DUV‐LED chips were flip‐chip bonded onto ceramic submounts coated with gold for light‐output testing.

## Conflict of Interest

The authors declare no conflict of interest.

## Supporting information

Supporting Information
