# Supplementary material for: Quasi‐2D Growth of Aluminum Nitride Film on Graphene for Boosting Deep Ultraviolet Light‐Emitting Diodes
Source: Adv Sci (Weinh). 2020 Jun 23;7(15):2001272. doi: 10.1002/advs.202001272 (PMC7404167; doi:10.1002/advs.202001272)
Supplement: Supplementary file 1 — Supporting Information [file ADVS-7-2001272-s001.pdf]

© 2020 Wiley-VCH GmbH

ADVANCED SCIENCE
Open Access

Supporting Information

for *Adv. Sci.*, DOI: 10.1002/advs.202001272

Quasi-two-dimensional Growth of AlN Film on Graphene for Boosting Deep Ultraviolet Light-emitting Diodes

Hongliang Chang, Zhaolong Chen, Bingyao Liu, Shenyuan Yang, Dongdong Liang, Zhipeng Dou, Yonghui Zhang, Jianchang Yan, Zhiqiang Liu, Zihui Zhang, Junxi Wang, Jinmin Li, Zhongfan Liu, Peng Gao, Tongbo Wei**

Copyright WILEY-VCH Verlag GmbH & Co. KGaA, 69469 Weinheim, Germany, 2020.

Supporting Information

Quasi-two-dimensional Growth of AlN Film on Graphene for Boosting Deep Ultraviolet Light-emitting Diodes

Hongliang Chang, Zhaolong Chen, Bingyao Liu, Shenyuan Yang, Dongdong Liang, Zhipeng Dou, Yonghui Zhang, Jianchang Yan, Zhiqiang Liu, Zihui Zhang, Junxi Wang, Jinmin Li, Zhongfan Liu, Peng Gao, Tongbo Wei**

Dr. H. L. Chang, Dr. D. D. Liang, Prof. J. C. Yan, Prof. Z. Q. Liu, Prof. J. X. Wang, Prof. J. M. Li, Prof. T. B. Wei
Research and Development Center for Semiconductor Lighting Technology, Institute of Semiconductors, Chinese Academy of Sciences, Beijing, 100083, China
Email: tbwei@semi.ac.cn

Dr. Z. L. Chen, Prof. Z. F. Liu
Center for Nanochemistry (CNC), Beijing Science and Engineering Center for Nanocarbons, College of Chemistry and Molecular Engineering, Peking University, Beijing, 100871, China

Dr. B. Y. Liu, Dr. Z. P. Dou, Prof. P. Gao
Electron Microscopy Laboratory, and International Center for Quantum Materials, School of Physics, Peking University, Beijing, 100871, China
Email: p-gao@pku.edu.cn

Prof. S. Y. Yang
State Key Laboratory of Superlattices and Microstructures, Institute of Semiconductors, Chinese Academy of Sciences, Beijing 100083, China

Dr. H. L. Chang, Prof. S. Y. Yang, Dr. D. D. Liang, Prof. J. C. Yan, Prof. Z. Q. Liu, Prof. J. X. Wang, Prof. J. M. Li, Prof. T. B. Wei
Center of Materials Science and Optoelectronics Engineering, University of Chinese Academy of Sciences, Beijing 100049, China

Dr. Z. L. Chen, Dr. B. Y. Liu, Dr. Z. P. Dou, Prof. Z. F. Liu
Beijing graphene institute (BGI), Beijing, 100095, P. R. China

Prof. Y. H. Zhang, Prof. Z. H. Zhang
School of Electronics and Information Engineering, Hebei University of Technology, Tianjin, 300401, China

Prof. P. Gao
Collaborative Innovation Center of Quantum Matter, Beijing 100871, China

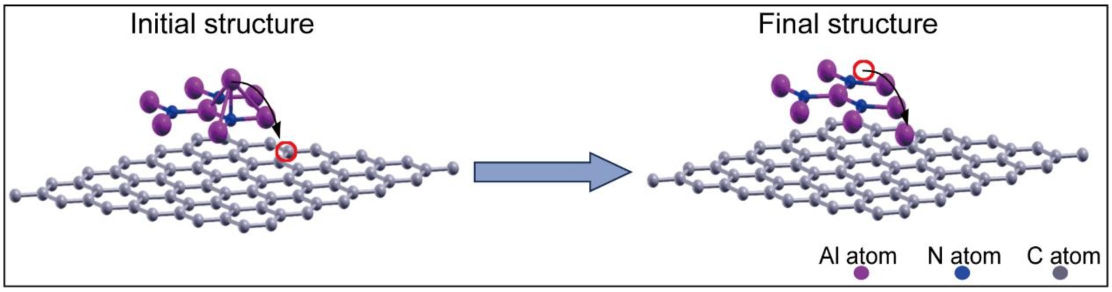

Figure S1. Adsorption of Al atom to the Al_7N_3 cluster on Gr sheet. In the initial structure, the Al atom is positioned on top of a N atom of the Al_7N_3 cluster (left). After relaxation, the Al atom jumps down and adsorbs on the Gr surface (right).

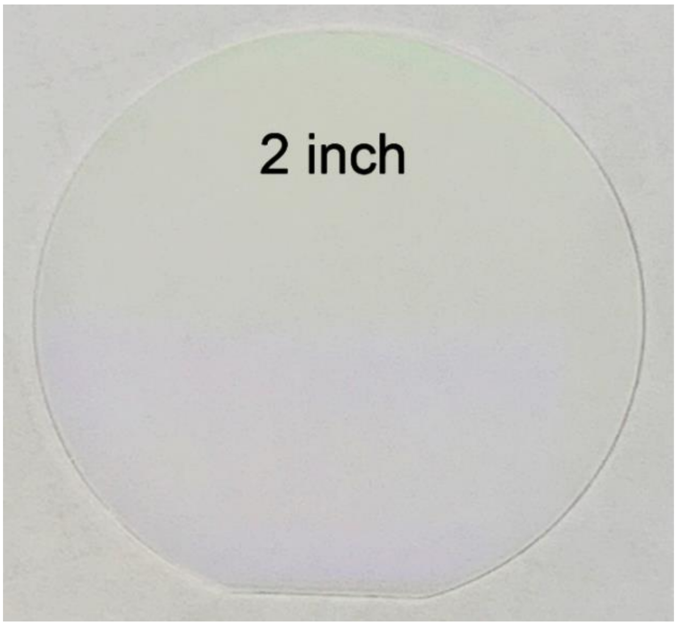

Figure S2. Photograph of an as-grown 2-inch Gr/NPSS wafer.

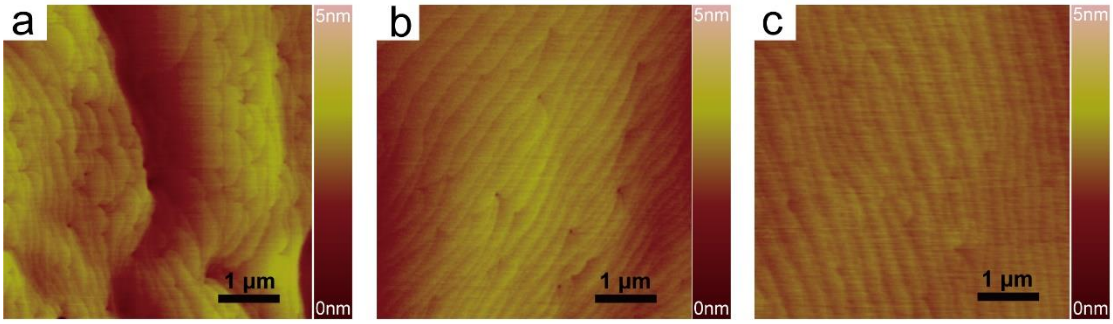

Figure S3. AFM characterizations of AlN films grown on NPSS with different processes. a, b, c) AFM images of the AlN film grown without buffer layer (a), with low-temperature (650 °C) AlN buffer (b) and with Gr buffer (c), respectively.

The root mean square (RMS) roughness of the films grown on NPSS without buffer layer, with the AlN LT buffer layer and with Gr buffer layer are measured by Atomic force microscope (AFM) to be 1.152, 0.233 and 0.142 nm, respectively (Fig. S3, Supporting Information), indicating that the surface of the film obtained in the presence of the Gr has the smoothest surface morphology.

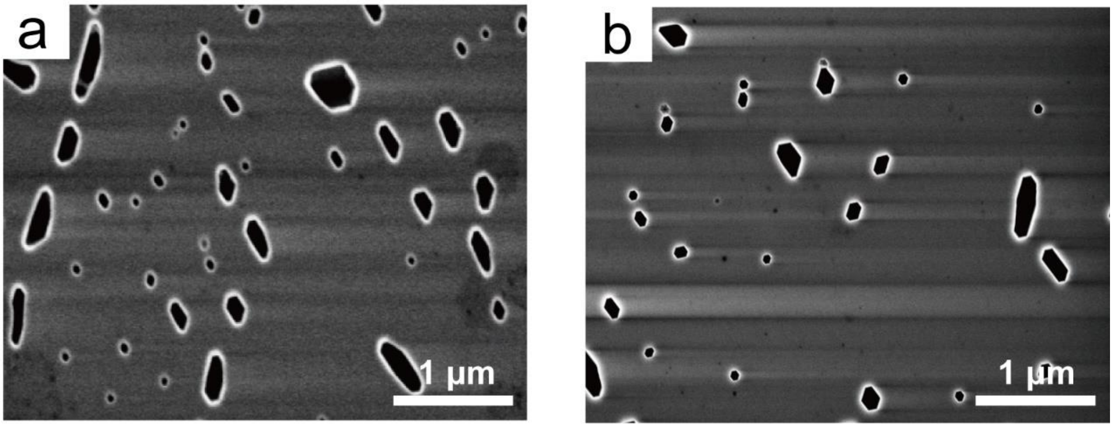

Figure S4. EPD analysis for crystal quality of AlN film grown on NPSS with different buffer layers. a, b) SEM images of the surface of wet etched AlN film grown with LT AlN buffer (a) and with Gr buffer (b), respectively.

To gain a deeper understanding of surface and crystalline quality, SEM is also used to observe the etch pit density (EPD) on AlN films with different buffer layers structures. The etching process is carried out in a H_2SO_4 and H_3PO_4 mixture solution with a 3:1 ratio at 270 °C for 4 min and the results are shown in Figs. S4, respectively.[1] The AlN film grown LT AlN buffer layer exhibits the higher EPD (1.7×10^8 cm^{-2}), while an obvious reduction of an EPD is observed for the AlN in the case of inserting a Gr buffer (8.3×10^7 cm^{-2}).

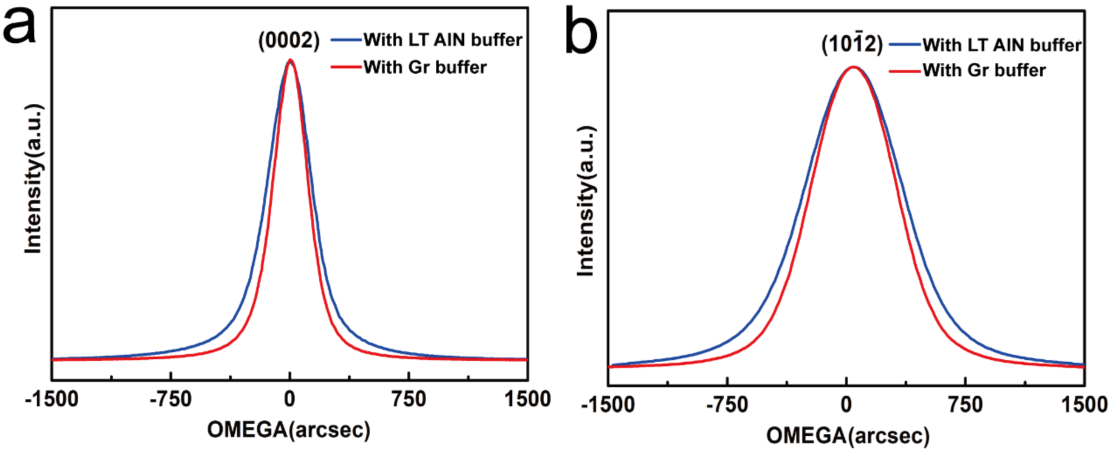

Figure S5. XRC analysis for crystal quality of AlN film grown on NPSS with different buffer layers. a, b) XRC of (a) (0002) and (b) $(10\bar{1}2)$ for AlN films grown with different buffer layers, respectively.

As depicted in Figure S5, compared with the (0002) full width at half maximum (FWHM) and $(10\bar{1}2)$ FWHM of X-ray rocking curve (XRC) of the AlN film grown on NPSS with the LT AlN buffer (327 and 555 arcsec, respectively), the FWHM of (0002) and $(10\bar{1}2)$ of the AlN film grown with Gr buffer (251 and 457 arcsec, respectively) are both greatly reduced. The corresponding estimated densities of screw and edge dislocations in the AlN epilayer on NPSS with LT AlN buffer are 2.34×10^{8} and 3.03×10^{9} cm^{-2}, respectively.[2] However, they are reduced to 1.55×10^{8} and 2.60×10^{9} cm^{-2} with the assistance of Gr buffer layer. Therefore, the addition of two different types of dislocation densities, we can know that the dislocation densities of AlN films with LT AlN and with Gr buffer are 3.264×10^{9} cm^{-2} and 2.755×10^{9} cm^{-2}, respectively, which further proves the better crystal quality of AlN film with Gr.

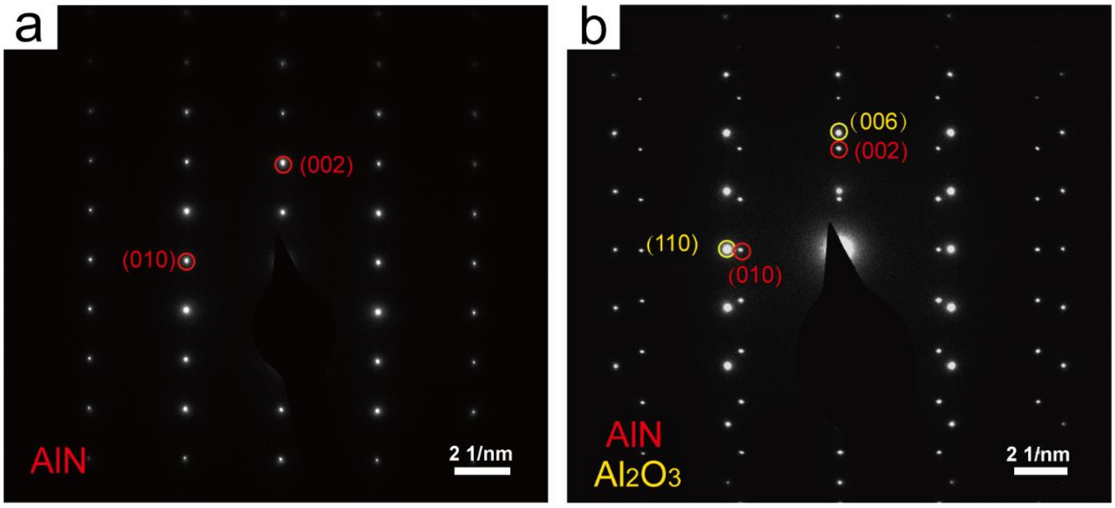

Figure S6. Selected-area electron diffraction (SAED) characterization of AlN and AlN/NPSS. a, b) SAED patterns taken from AlN layer (a) and the interface between AlN and Gr/NPSS (b).

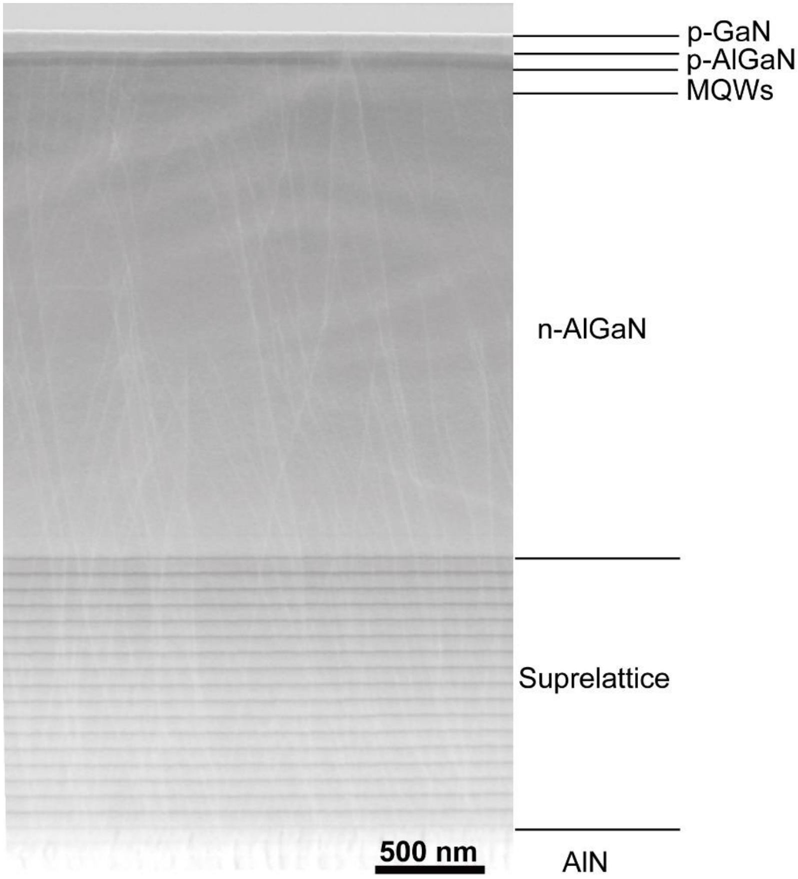

Figure S7. Cross-sectional STEM image of as-grown DUV-LED with Gr.

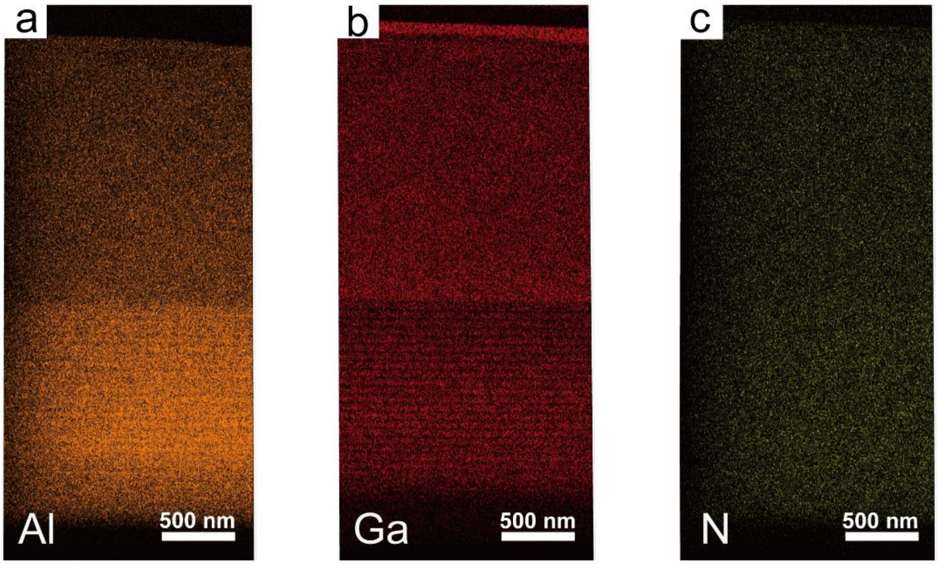

Figure S8. Energy dispersive X-ray spectroscopy (EDS) characterization of as-grown DUV-LED with Gr. a, b, c) EDS mapping of Al (a), Ga (b), and N (c) elements of as-grown DUV-LED on AlN/Gr/NPSS.

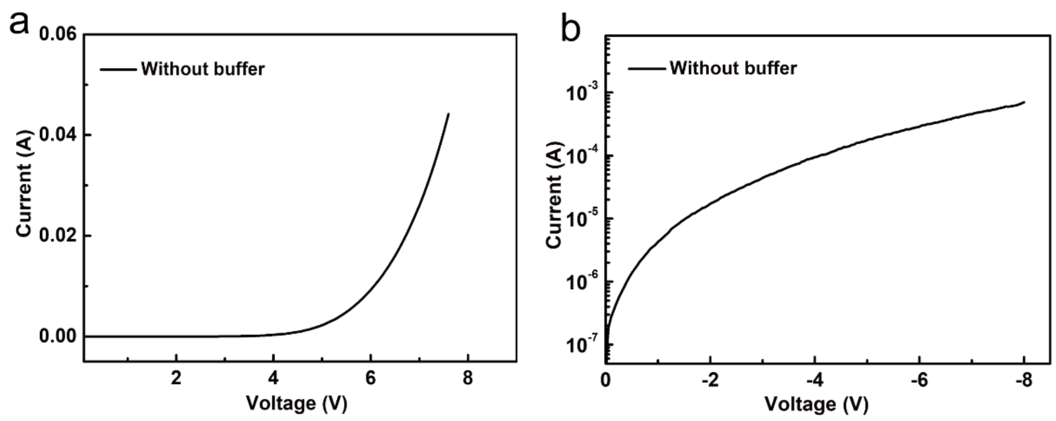

Figure S9. Current-voltage characteristics of as-fabricated DUV-LEDs without buffer layer. a) Current-voltage curve of DUV-LED without buffer. b) Reverse current-voltage curve on a semi-log scale.

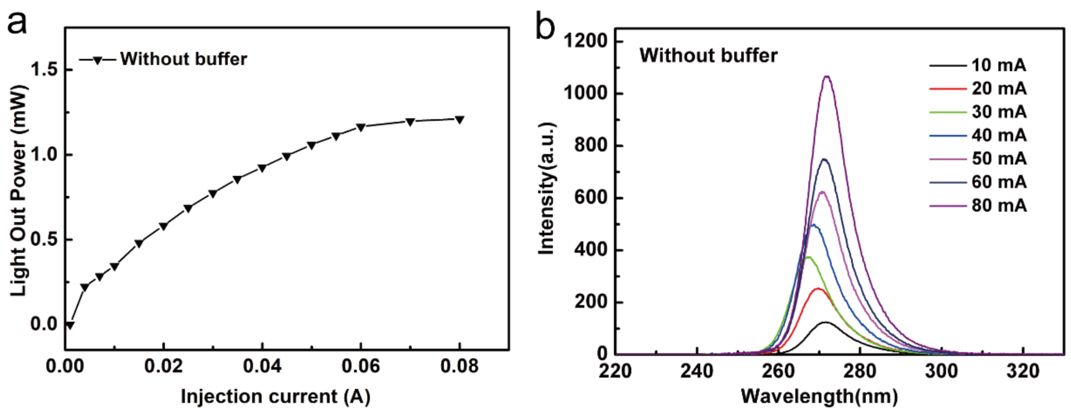

Figure S10. Electroluminescence of as-fabricated DUV-LEDs without buffer layer. a) The light output power of the as-fabricated DUV-LEDs without buffer as a function of injection current. b) EL spectra of DUV-LEDs measured by varying the injection current from 10 to 80 mA.

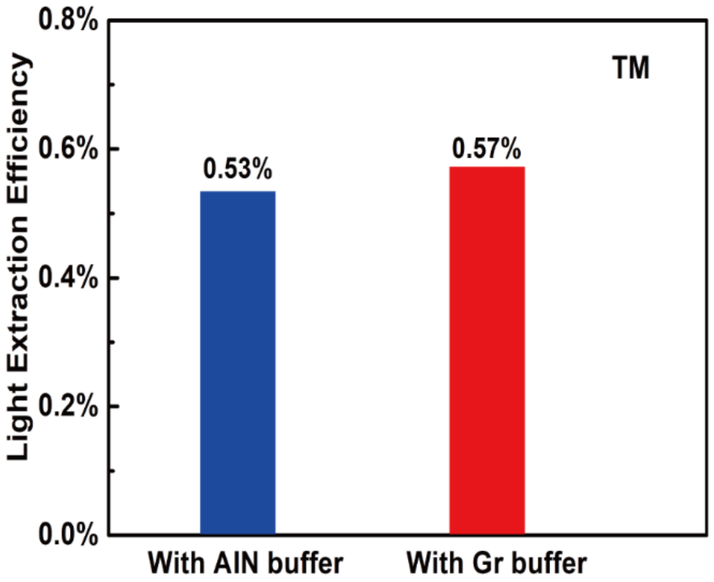

Figure S11. FDTD simulations characterization of the LEE of DUV-LEDs with different buffer layers for TM polarizations.

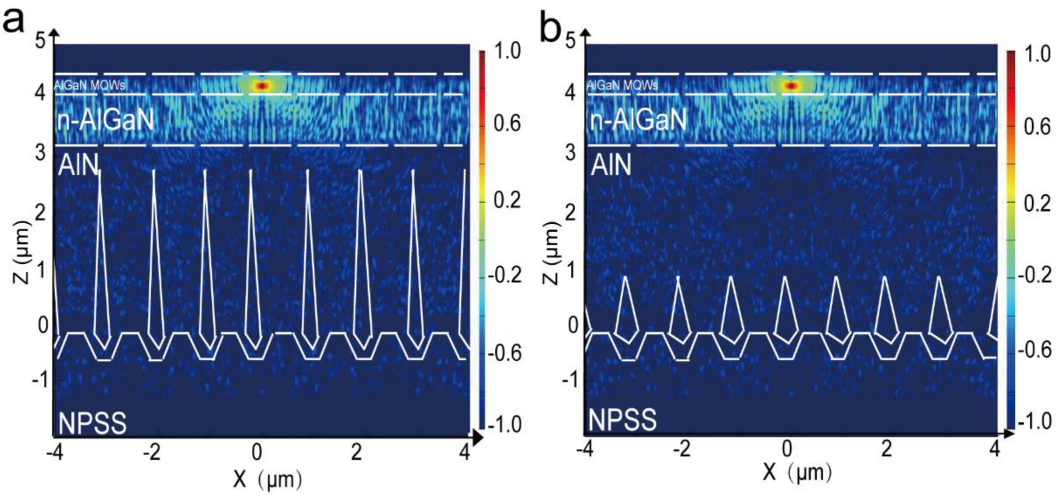

Figure S12. FDTD simulation characterization of as-fabricated DUV-LEDs. a, b) Cross-sectional electric field distributions of the DUV-LEDs (a) with LT buffer and (b) with Gr buffer for TM polarizations at the x–z plane, respectively.

Reference

[1] T. Wei, X. Ji, K. Wu, H. Zheng, C. Du, Y. Chen, Q, Yan, L. Zhao, Z. Zhou, J. Wang, J. Li, *Opt. Lett.* **2014**, 39, 379.

[2] H. Heinke, V. Kirchner, S. Einfeldt, D. Hommel, *Appl. Phys. Lett.* **2000**, 77, 2145.
